# A theoretical framework for quantifying blood volume flow rate from dynamic angiographic data and application to vessel-encoded arterial spin labeling MRI^☆^

**DOI:** 10.1016/j.media.2013.06.005

**Published:** 2013-12

**Authors:** Thomas W. Okell, Michael A. Chappell, Peter Jezzard

**Affiliations:** a Centre for Functional Magnetic Resonance Imaging of the Brain, Nuffield Department of Clinical Neurosciences, University of Oxford, Oxford, UK; b Institute of Biomedical Engineering, Department of Engineering Science, University of Oxford, Oxford, UK

**Keywords:** Blood volume flow rate quantification, Magnetic resonance imaging, Dynamic angiography, Vessel-encoded pseudocontinuous arterial spin labeling, Cerebral arteries

## Abstract

Angiographic methods can provide valuable information on vessel morphology and hemodynamics, but are often qualitative in nature, somewhat limiting their ability for comparison across arteries and subjects. In this work we present a method for quantifying absolute blood volume flow rates within large vessels using dynamic angiographic data. First, a kinetic model incorporating relative blood volume, bolus dispersion and signal attenuation is fitted to the data. A self-calibration method is also described for both 2D and 3D data sets to convert the relative blood volume parameter into absolute units. The parameter values are then used to simulate the signal arising from a very short bolus, in the absence of signal attenuation, which can be readily encompassed within a vessel mask of interest. The volume flow rate can then be determined by calculating the resultant blood volume within the vessel mask divided by the simulated bolus duration. This method is applied to non-contrast magnetic resonance imaging data from a flow phantom and also to the cerebral arteries of healthy volunteers acquired using a 2D vessel-encoded pseudocontinuous arterial spin labeling pulse sequence. This allows the quantitative flow contribution in downstream vessels to be determined from each major brain-feeding artery. Excellent agreement was found between the actual and estimated flow rates in the phantom, particularly below 4.5 ml/s, typical of the cerebral vasculature. Flow rates measured in healthy volunteers were generally consistent with values found in the literature. This method is likely to be of use in patients with a variety of cerebrovascular diseases, such as the assessment of collateral flow in patients with steno-occlusive disease or the evaluation of arteriovenous malformations.

## Highlights

• We propose a method for dynamic angiographic blood volume flow rate quantification.
• This technique is compatible with most types of dynamic angiographic data.
• We applied this method to vessel-encoded arterial spin labeling angiography.
• Blood volume flow rate estimates were validated using a flow phantom.
• Results in the cerebral arteries of healthy volunteers agree with previous studies.

## Graphical abstract

**Figure:**
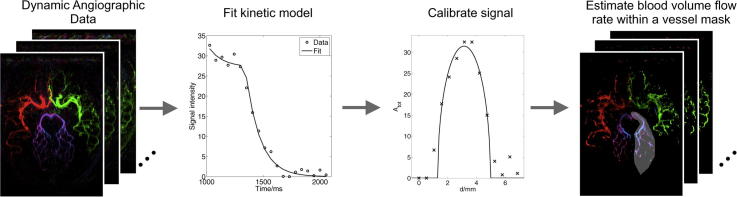

## 1 Introduction

Angiographic methods, which generate images of blood vessels, are of great importance in the assessment of vascular diseases, such as atherosclerosis. They can provide information on vessel morphology and function which aids clinicians with diagnosis, prognosis and treatment planning in these patients. Vessel-selective angiography provides additional information about the relative importance of each feeding artery which is useful in a number of areas, such as the assessment of collateral blood flow or the evaluation of blood supply to a tumor or arteriovenous malformation. However, many angiographic methods provide only qualitative information on blood flow, making objective comparison across arteries and across subjects difficult.

One method that overcomes this restriction is phase-contrast Magnetic Resonance Angiography (MRA) (e.g. Dumoulin and Hart, 1986) which provides quantitative blood velocity and flow rate measurements. However, it is somewhat hampered by long scan times (Miyazaki and Lee, 2008) and by a lack of information regarding the arterial source of downstream blood flow. To achieve blood flow rate quantification from other types of dynamic angiographic data a simple modification of perfusion-based single voxel models (such as Buxton et al., 1998) is not appropriate since there is no flow-dependent accumulation of labeled blood in large vessels. Indeed, the signal contrast measured in these experiments depends directly on blood volume, not blood flow.

Flow rate quantification from dynamic angiographic MRI data using a pulsed arterial spin labeling (ASL) preparation has previously been reported (van Osch et al., 2006), but their method requires an extra calibration scan to measure the arterial input function (AIF). Measurement of the absolute AIF is not possible for 2D projection methods such as X-ray digital subtraction angiography (DSA). In addition, for techniques in which imaging cannot commence until the end of a long labeling period, such as those based on continuous ASL (CASL: Williams et al., 1992) or pseudocontinuous ASL (PCASL: Dai et al., 2008), measurement of the early part of the AIF is precluded. This is unfortunate, since these preparations can produce longer boluses of labeled blood than pulsed ASL, improving signal-to-noise ratio (SNR). In addition, vessel-selective labeling with pulsed ASL (Hendrikse et al., 2004; Golay et al., 2005) is restricted to arteries over which a single slab can be positioned, excluding all other vessels. In CASL or PCASL, this restriction does not apply since vessel-selective labeling is performed within a single plane. Such approaches have recently been applied to angiographic imaging by a number of groups (Okell et al., 2010; Robson et al., 2010; Helle et al., 2011), but these studies have been limited to qualitative depictions of flow and the measurement of simple timing parameters.

In this study we propose a framework for absolute blood volume flow rate quantification using generic dynamic angiographic data. This involves the fitting of a kinetic model in each voxel, accounting for relative blood volume, delayed blood arrival, bolus dispersion and signal attenuation. A self-calibration method is described for relating the relative blood volume parameter to absolute blood volume in either 2D or 3D acquisitions, using the same data set. Using these results a method for quantifying blood volume flow rate by simulating a very short bolus of labeled blood is described.

Here we apply this methodology to dynamic 2D MRI data acquired with a pulse sequence (Okell et al., 2010) which combines a vessel-encoded PCASL (VEPCASL) preparation (Wong, 2007) with a segmented spoiled gradient echo readout (Günther et al., 2002; Sallustio et al., 2008) for which a specific kinetic model describing the signal evolution in a single voxel has recently been described (Okell et al., 2012). This sequence has the advantages of not requiring an invasive procedure, the administration of a contrast agent or exposure to ionizing radiation. However, in principle the proposed method is applicable to any dynamic angiographic imaging modality for which the time course of the relative concentration of labeled blood in proximal arteries is known or can be measured and attenuation of the labeled blood signal can be described. Validation of this method using a flow phantom is presented, along with results of its application in healthy volunteers.

## 2 Theory

### 2.1 General approach

**Fig. 1:**
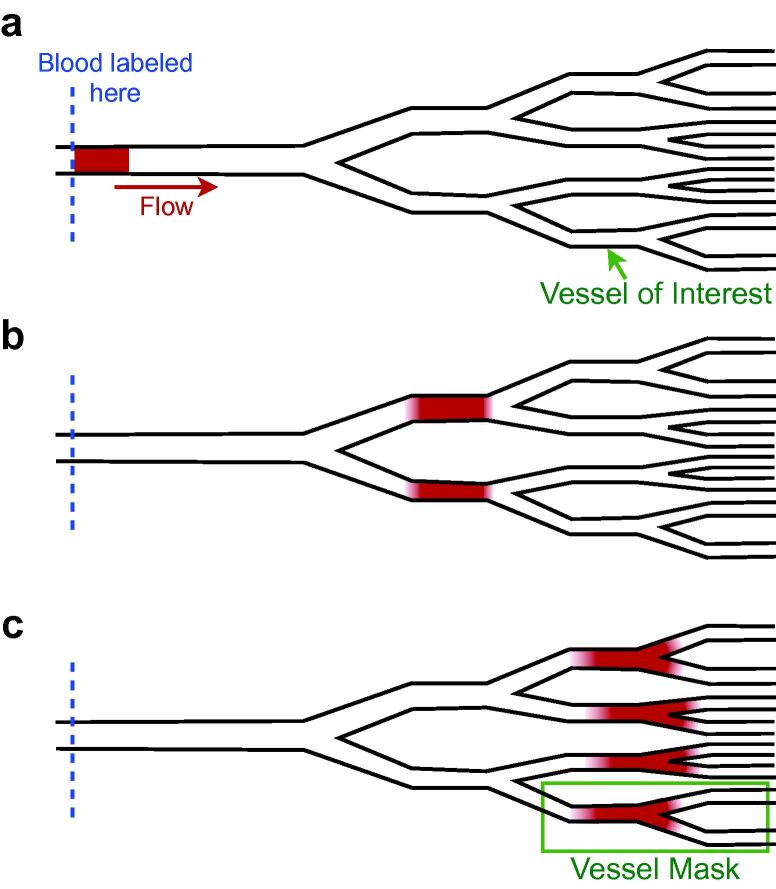
Schematic showing the general approach to flow rate quantification: (a) a short, uniform bolus of labeled blood with duration *τ* is created in a proximal vessel; (b) the bolus travels into the vessel network, splitting according to the flow ratios between different branches, and begins to disperse; and (c) the bolus arrives in the vessel of interest. If the spatial extent of the bolus, including dispersion, is small enough to be encompassed within a mask drawn over the vessel of interest and any downstream branches, then the volume of labeled blood which has flowed into this vessel can be estimated. This volume of blood is generated during time *τ*, and must therefore flow into the vessel of interest during every time period, *τ*, if the flow rate is constant. The blood volume flow rate into the vessel of interest can therefore be calculated by taking the volume of labeled blood within the vessel mask and dividing by *τ*.

Consider a dynamic angiography experiment (as illustrated in Fig. 1) using a rect-shaped bolus of duration, *τ*, where all of the blood flowing through a defined point in the upstream vessels is perfectly labeled. Assuming that there is no flow pulsatility and that the subject’s physiology remains constant, the volume of blood which is labeled must flow through the vessel network every time period *τ*. By extension, the volume of this labeled blood which flows into any downstream vessel must also flow through that vessel during every time period *τ*. Even if the bolus disperses during its passage, by the conservation of mass the volume of labeled blood flowing into the vessel will not be affected. Therefore, the blood volume flow rate within a downstream vessel can be calculated simply as the volume of labeled blood which flows into it divided by the bolus duration, *τ*.

In order to be able to accurately estimate the volume of labeled blood which ends up in a vessel of interest, the entire bolus of labeled blood must be present in that vessel, or its downstream branches, at one point in time. To achieve this, a very short bolus duration can be used to ensure that the spatial extent of the bolus is small. If this is sufficient to ensure that the entire bolus is present in the vessel of interest at one point in time, even in the presence of dispersion, and the total volume of labeled blood within this vessel can be estimated from the measured signal intensity, then this should be sufficient to estimate the blood volume flow rate within that vessel. However, to perform such an experiment would be difficult: in some angiographic modalities a rect-shaped bolus with uniform labeling cannot be achieved, the use of a short labeling duration leads to poor SNR (Okell et al., 2010) and decay of the signal during imaging would further confound the measurement.

**Fig. 2:**
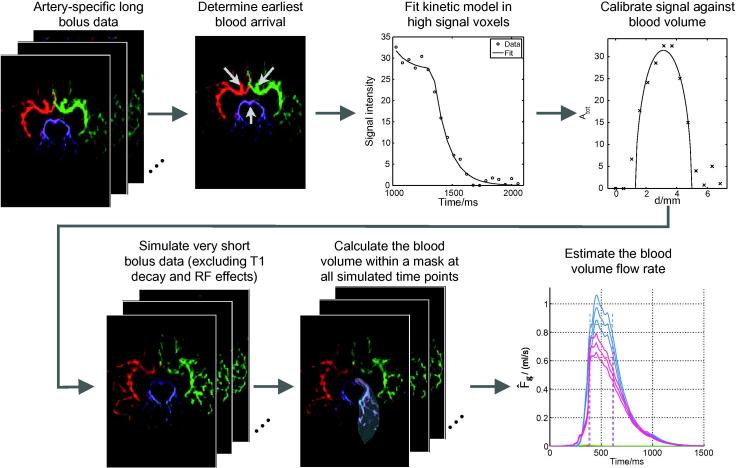
Overview of the processes undertaken to obtain quantitative blood volume flow rate estimates from dynamic angiographic data, illustrated with VEPCASL images. Here and elsewhere color is used to represent the origin of the blood signal: red = RICA, green = LICA, blue = RVA, purple = LVA. (For interpretation of the references to colour in this figure legend, the reader is referred to the web version of this article.)

To overcome these problems, we use a kinetic model which describes the time evolution of the signal in a single voxel from a given feeding artery, incorporating blood volume, arrival time, bolus dispersion and signal attenuation. This model can be fitted to high SNR data acquired using a long bolus duration. Once the model parameters in each voxel have been estimated, the theoretical signal arising from a more ideal very short bolus experiment can be simulated over an arbitrary range of time points, and with the confounding signal attenuating effects removed. After signal calibration, the blood volume within a vessel of interest can be calculated, and thus the blood volume flow rate can be determined. Fig. 2 outlines the processing stages required for this approach.

### 2.2 Kinetic model

The derivation of a kinetic model to describe the signal evolution in a single voxel for dynamic angiography data based on a CASL/PCASL preparation and a spoiled gradient echo readout has been previously reported (Okell et al., 2012). This model can be generalized to any dynamic angiographic data as follows. The signal, *S*, measured in a single voxel at time *t* will depend upon the relative concentration of the tracer in a proximal vessel, *c*_in_. This tracer takes a time Δ*t* to arrive at the voxel. An additional delay, *t*′, due to dispersion is modeled by convolution with a dispersion kernel, *D*(*t*′). For clarity, the variables used in this paper are listed in Table 1. Note that we have substituted some variable symbols here from Okell et al. (2012) to maintain consistency with other literature.

Signal attenuation due to tracer decay and resulting from the imaging method are described by multiplicative factors *T* and *Ψ*, respectively. The measured signal is also scaled by the volume of blood within the voxel, *v*, and a calibration factor, *S*_0_, which relates blood volume to measured signal in the absence of signal attenuation, which we combine into a single scaling factor *A* = *S*_0_*v*. Thus, the measured signal, *S*, is given by:

(1) $$S(t)=A\int_{-\infty }^{+\infty }c_{\mathrm{in}}(t-t^{\prime }-\Delta t)\;D(t^{\prime })\;T(\Delta t\text{,}t^{\prime }\text{,}t)\;\Psi (\Delta t\text{,}t^{\prime }\text{,}t)\;{\mathrm{dt}}^{\prime }$$

This equation assumes the measured signal is proportional to the volume of the tracer within each voxel. For imaging modalities where this is not the case an appropriate conversion would have to be performed either as a preprocessing step or incorporated into the kinetic model. Note that Eq. (1) can be used to fit data from multiple feeding arteries whose signals have been separated in preprocessing. The fit to the signal from artery *j* gives *A*_*j*_ = *S*_0_ *v*_*j*_ where *v*_*j*_ is the volume of blood within the voxel originating from artery *j*.

In the case of CASL/PCASL angiography *c*_in_ is a rect function with duration equal to the labeling duration, *τ*:

(2) $$c_{\mathrm{in}}(t)=\left\{\begin{array}{cc} 0 & t<0\\ 1 & 0⩽t<\tau \\ 0 & t⩾\tau \end{array}\right)$$

Note that although the relative concentration of labeled water takes a value of one here, this does not imply that the labeling efficiency is 100%. A reduced labeling efficiency would result in a global reduction of the measured signal, which is incorporated into the calibration factor, *S*_0_.

For the VEPCASL data used in this study we chose a gamma variate model for *D* characterized by sharpness, *s*, and time to peak, *p*. In this case *T* represents decay of the label due to *T*_1_ relaxation and *Ψ* represents signal attenuation due to the imaging radio frequency (RF) pulses. Note that *Ψ* depends on the time at which blood first arrives in the imaging region, Δ*t*_min_, which is determined as an explicit step in preprocessing (described below) rather than being fitted from the data. This leaves four free parameters in each voxel: *A*, Δ*t*, *s* and *p*. The explicit mathematical forms for *D*, *T* and *Ψ* relevant for VEPCASL angiography can be found in our previous paper (Okell et al., 2012).

### 2.3 Calibration

The kinetic model described above allows only relative blood volume to be determined, and hence only relative blood volume flow rate. In order to calculate absolute blood volume flow rates, the calibration factor *S*_0_ must be determined. If a 3D angiography acquisition were performed, and provided one or more voxels small enough to fit entirely within an artery could be found, then this should be sufficient for the calibration. Fitting Eq. (1) to such voxels would yield an estimate of *A* for each feeding artery, the sum of which we define as *A*_tot_. In these voxels the sum of *v* over all feeding arteries must be equal to the voxel volume Δ*x* Δ*y*Δ*z*, so we can write:

(3) S0^≈AtotΔxΔyΔzvoxelswithinarteries

**Fig. 3:**
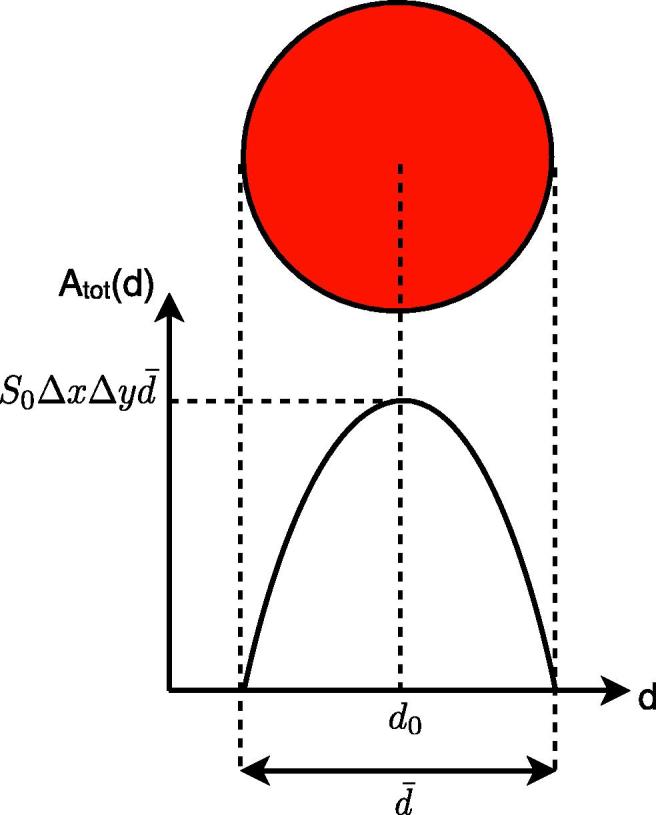
Vessel geometry used to estimate the calibration factor, *S*_0_. Assuming a circular vessel cross-section, *A*_tot_ varies predictably as a function of distance across the vessel, *d*, with the shape of the curve depending only on *S*_0_, the position of the vessel center, *d*_0_, the vessel diameter, $\overset{¯}{d}$, and the voxel dimensions, Δ*x* and Δ*y*.

In a 2D acquisition, where a thick slab is imaged to yield a 2D projection of the vascular network, the volume of blood leading to the measured signal is unknown since it only occupies a portion of the slab thickness. However, if a vessel can be identified that runs parallel to the imaging plane, and if it is assumed that the cross section of that vessel is approximately circular, then measurements of vessel diameter, $\overset{¯}{d}$, within the imaging plane can be used to estimate the volume of blood producing the measured signal and therefore *S*_0_. For example, plotting a profile of *A*_tot_ against distance, *d*, perpendicular to the vessel direction (see Fig. 3) allows a fit to both $\overset{¯}{d}$ and *S*_0_ simultaneously by considering the equation for a circle, to give:

(4) $$A_{\mathrm{tot}}(d)=\left\{\begin{array}{cc} S_{0}\Delta x\Delta y\sqrt{{\overset{¯}{d}}^{2}-4{\left(d-d_{0}\right)}^{2}} & |d-d_{0}|⩽\overset{¯}{d}/2\\ 0 & |d-d_{0}|>\overset{¯}{d}/2 \end{array}\right)$$

where *d*_0_ is the location of the center of the vessel.

### 2.4 Flow rate quantification within a vessel segment

The volume flow rate of blood, *F*_*g*_, in a given vessel, *g*, is given by the volume of blood passing through it per unit period of time. As discussed above, under constant flow rate conditions, the volume of labeled blood which flows into this vessel, *V*_*g*_, arises from experimentally imposed labeling for a time *τ*, and thus must flow into this vessel during every time interval, *τ*. Therefore, the volume flow rate through this vessel can be written as:

(5) $$F_{g}=\frac{V_{g}(\tau )}{\tau }$$

As mentioned previously, in order to get an accurate estimate of *V*_*g*_, the labeled bolus must be entirely contained within vessel *g* and the imaging region. In addition, signal attenuating effects must not be present. This can be achieved by using the parameter estimates derived from fitting the kinetic model described above to the data in order to simulate the signal, *S*′, in each voxel that would have arisen from a very short labeling duration, *τ*′, in the absence of signal attenuating effects (i.e. *T* = *Ψ* = 1):

(6) $$S^{\prime }(t)=A\int_{t-\Delta t-\tau^{\prime }}^{t-\Delta t}D(t^{\prime }\text{,}s\text{,}p)\;{\mathrm{dt}}^{\prime }$$

Summing this simulated signal across a mask encompassing vessel *g* (which may include its downstream branches) and dividing by the calibration factor *S*_0_ gives an estimate of the labeled blood volume within the vessel at the specified time. For times during which the simulated bolus lies entirely within the mask and imaging region, the volume flow rate can be estimated as:

(7) F^g=1τ′∑vesselmaskS′(t)S0

**Fig. 4:**
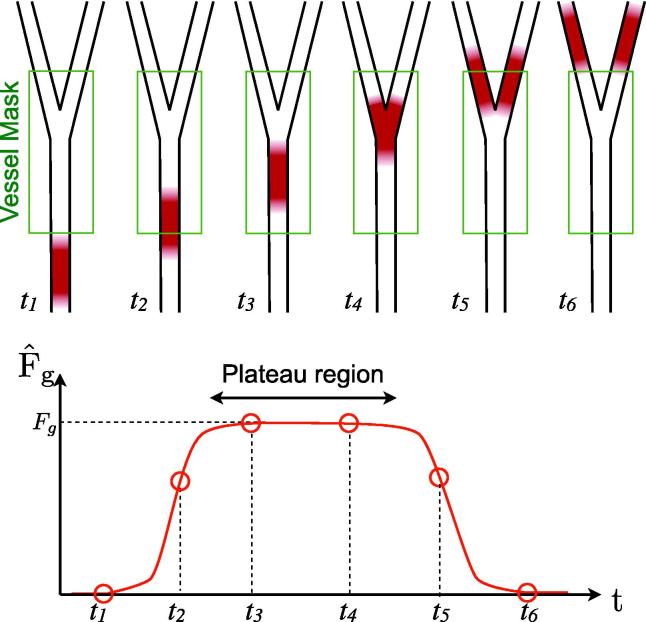
Schematic depiction of the expected variation in estimated blood volume flow rate, F^g, within a vessel mask as a function of simulated time. The top row shows a simulated bolus at a number of time points flowing through a vessel and its downstream branches, over which a mask has been drawn. At each time point the labeled blood volume within the mask is calculated and divided by *τ*′ to give F^g. While the simulated bolus lies completely within the vessel mask (t=t3,t4),F^g levels out to a constant value (the “plateau” region) equal to the true flow rate, *F*_*g*_. Note that even though the vessel branches within the mask, the volume of labeled blood is constant so this does not affect the flow rate quantification.

*F*_*g*_ should be independent of the time at which it is measured. Therefore, for any *t* where the labeled bolus is still located within the vessel mask and has not moved out of the imaging slab or started exchanging with tissue, the same volume flow rate should be calculated. Calculating F^g at multiple time points thus provides an opportunity to test the model and obtain a more robust estimate. Fig. 4 shows schematically the expected variation in the estimated blood volume flow rate within a vessel mask over a range of simulated time points.

It is noted that in cases where dispersion is very significant or where the vessel segment of interest is small, the simulated bolus may not lie entirely within the vessel mask and imaging slab at any of the simulated time points. In such cases the volume flow rate will be underestimated.

## 3 Materials and methods

All experiments performed for this study were undertaken using a 3 Tesla Siemens MRI scanner (both TIM Verio and TIM Trio, Siemens Healthcare, Erlangen, Germany) using a 12 channel head coil for signal reception and body coil for transmission.

### 3.1 Flow phantom validation

A flow phantom was used to validate the proposed method by allowing comparison between known volume flow rates and those estimated using the method described above. The phantom consisted of a variable speed water pump (Ismatec SA, Zürich) connected to a long rubber tube with an internal diameter of approximately 4 mm. Two sections of this tube were secured to a foam pad inside the scanner bore with water running in opposite directions in the two sections. This foam pad was placed within the head coil along with a water bottle phantom to ensure appropriate coil loading.

A 3D time-of-flight (TOF) sequence was used for phantom localization and planning of the vessel-encoded scans. An additional TOF scan was acquired using the body coil for signal reception to allow correction for the variation in the head coil sensitivity across the field of view. 2D time-resolved VEPCASL angiography data were acquired as described by Okell et al. (2010) (voxel size 1.1 × 1.1 mm interpolated to 0.55 × 0.55 mm, slab thickness 50 mm, temporal resolution 55 ms, 20 time frames) with the exception that only four encoding cycles were necessary to separate the signal from the two tubes: non-selective tag, non-selective control, tag right tube whilst controlling left and tag left tube whilst controlling right. Acquisitions were performed over a range of flow phantom velocities. The true volume flow rate was measured during each acquisition by recording the time taken for the outlet tube to fill a measuring beaker to a fixed volume. This was repeated three times and the fixed volume divided by the average time taken to give the volume flow rate in ml/s.

### 3.2 Healthy volunteer experiments

Six healthy volunteers with no known neurological disease (4 male, age range 24–39; mean age 32) were recruited and scanned under a technical development protocol agreed with local ethics and institutional committees. A 3D TOF sequence was used for planning the vessel-encoded acquisitions. Six-cycle vessel-encoded dynamic angiography acquisitions (as per Okell et al., 2010) in transverse (50 mm imaging slab) and coronal (100 mm imaging slab) views centered on the circle of Willis were also performed. A sagittal acquisition was not performed due to overlap of vessels in this view. These source data have been used in previous publications (Okell et al., 2010; Okell et al., 2012).

### 3.3 Data preprocessing

Preprocessing was performed as per Okell et al. (2012), including separation of vascular components using a maximum *a posteriori* (MAP) Bayesian solution (method BT3 Chappell et al., 2012) to the general framework for vessel-encoded analysis (Chappell et al., 2010), phase correction to convert the complex signals to real signals, correction for coil non-uniformity, determination of earliest blood arrival in the imaging region (Δ*t*_min_) and fitting of the kinetic model in high signal voxels for each feeding artery using a Bayesian framework.

The generation of the high signal mask was modified slightly for data in the coronal view (including for the flow phantom data) since it was noted that in very proximal vessels the blood washes out very rapidly, leading to a low mean signal and therefore exclusion from the mask. To overcome this limitation another mask was created in the same manner using the maximum, rather than the mean, signal over time, and the intersection of these two masks was used for the fitting.

Pre-processing of the flow phantom and healthy volunteer data differed slightly: for the flow phantom, the signal was separated into contributions from water originating from the two tubes plus static water. In healthy volunteers, the signal was separated into contributions from static tissue plus the four main brain feeding arteries: namely the right and left internal carotid arteries (RICA and LICA) and vertebral arteries (RVA and LVA). In addition, correction for coil non-uniformities in the flow phantom data was performed using a body coil image as a reference, since the segmentation approach taken originally for human subjects is not appropriate for such a phantom.

### 3.4 Calibration

Since the experimental data collected in this study consisted of 2D projections from thick slabs it was necessary to determine the blood calibration factor using the 2D method described above. Once the kinetic model fitting was complete a map of the parameter *A*, which is proportional to blood volume, was used to calibrate the signal. First, *A* was summed across all feeding arteries to give *A*_tot_, which is proportional to the total blood volume within each voxel. The calibration factor, *S*_0_, was then determined by fitting Eq. (4) to a profile of *A*_tot_ through ten manually-selected vessel segments. The TOF images were used to ensure that the chosen vessel segments were running approximately parallel to the imaging plane. *S*_0_ was then taken as the average of these measurements. In the transverse acquisitions this corresponded to three profiles through both left and right proximal middle cerebral arteries (M1 segments) and two profiles through both left and right proximal posterior cerebral arteries (P2 segments). In coronal acquisitions, four profiles were drawn through both left and right internal carotid arteries (two through the C1 segments and two through the C3 segments, as defined by Bouthillier et al. (1996)) and two through the distal basilar artery. To account for uncertainty in *S*_0_ the standard error of its mean value was calculated. This uncertainty was propagated through to the flow rate quantification stage.

### 3.5 Flow rate quantification

Volume flow rate quantification was performed by simulating the signal, *S*′, from a very short bolus duration (*τ*′ = 1 ms) in the absence of *T*_1_ decay and RF effects (using Eq. (6)). Note that the choice of *τ*′ should not have a significant impact on the flow rate quantification if it is small relative to dispersion of the bolus (i.e. *p* and 1/*s*). In our experiments in the cerebral vessels 1/*s* is on the order of 100 ms. To ensure that the measured volume flow rates were not dependent on the choice of *τ*′, the processing of the flow phantom data was performed at a range of *τ*′ values.

In order to propagate uncertainties in the fitting procedure through to the flow rate quantification a numerical estimate of the covariance matrix associated with the parameters was recorded as part of the fitting procedure (Okell et al., 2012). The parameter covariance matrix was used to determine a grid of points in parameter space over which the mean and variance of the simulated signal, *S*′, were calculated, weighted by the true posterior probability of the parameters given the data.

As shown in Fig. 4 the estimated volume flow rate within a given vessel mask is expected to plateau during the time that the bolus is entirely within the mask. To estimate this plateau region the approximate start and end times of the bolus passage through the mask were determined by thresholding the estimated volume flow rate at 20% of its maximum. Assuming the plateau occupies a significant proportion of this broad peak, the median value within it should be approximately equal to the volume flow rate within the plateau region and should also be relatively insensitive to noisy peaks and troughs in the curve. The plateau region was defined as the interval bounded by the times at which the estimated volume flow rate first and last exceeds this median value. The final estimated flow rate and its error were calculated as the mean and standard deviation within this plateau region.

**Fig. 5:**
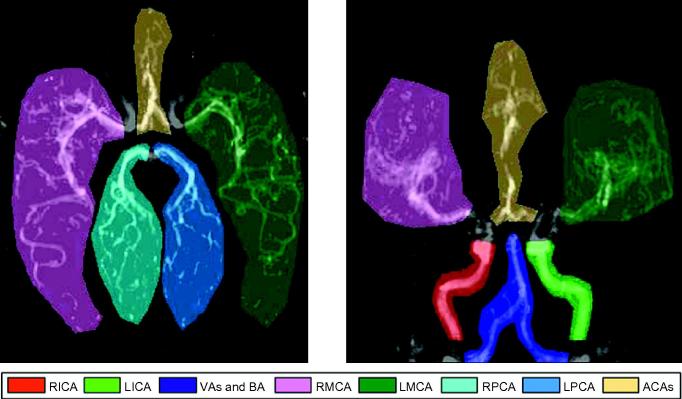
Manually drawn masks used for flow rate quantification in the major cerebral arteries overlaid on a map of *A*_tot_ in a healthy volunteer in both transverse and coronal views. Note that the ACAs are combined due to their proximity, the VAs and BA are analysed as one to ensure a large enough mask is used and the PCAs are not analysed in the coronal view due to overlap with other vessels.

Quantification was performed within a number of masks which were drawn manually for each data set (examples shown in Fig. 5). In the transverse view these encompassed the right and left middle cerebral arteries (RMCA and LMCA) and posterior cerebral arteries (RPCA and LPCA) along with both anterior cerebral arteries (ACAs), which were considered together due to their proximity. In the coronal view the MCAs and ACAs were also analysed, but the more proximal coverage also allowed flow quantification in the right and left internal carotid arteries (RICA and LICA) and the combined flow in the vertebral and basilar arteries (VAs and BA). This larger mask ensured that the whole simulated bolus was contained within it. Note that the PCAs were not analysed in the coronal view as they overlap considerably with other vessels, rendering attempts at quantification inaccurate.

## 4 Results

### 4.1 Flow phantom validation

**Fig. 6:**
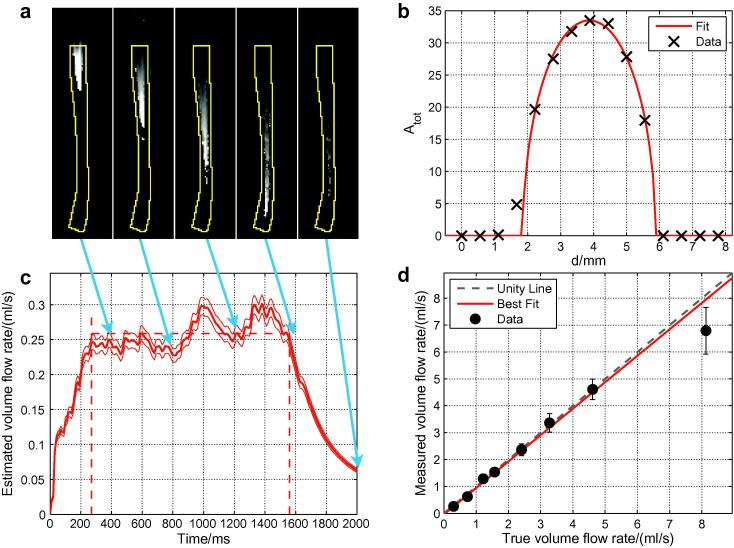
Validation of the quantification method in a flow phantom: (a) a series of images of the simulated signal, *S*′, (with the vessel mask outline overlaid in yellow) at different simulated time points (indicated by the arrows); (b) an example of the calibration method showing the fit of Eq. (4) to a profile of *A*_tot_ across one of the tubes; (c) the estimated volume flow rate at each time point (thick line) with error margins (thin lines) and the estimated plateau region (dashed lines); and (d) the volume flow rate and its error calculated using the proposed method plotted against the true volume flow rate as measured during the experiment. Results for flow rates below about 4.5 ml/s are very close to the ideal unity line. The line of best fit has gradient 0.98 ± 0.03 and intercept −0.04± 0.02 ml/s. Note that (a) and (c) were generated using data where the true flow rate was 0.30 ml/s.

Fig. 6 shows the results of the flow phantom experiment. The simulated signal behaves as expected with the bolus travelling steadily downstream (flowing from the top of the figure to the bottom) and becoming more dispersed over time. The calibration method appears to work well with Eq. (4) providing a good fit to the data. As predicted (see Fig. 4) the estimated volume flow rate reaches a plateau before falling at later simulated time points.

The volume flow rate estimated using the proposed method is very close to the true flow rate measured from the phantom outflow pipe during the experiment. The highest measured flow rate was somewhat underestimated, but the line of best fit (*χ*^2^ = 4.0) suggests that on average that the systematic underestimation of the volume flow rate is only 2 ± 3% across the measured range.

As expected, the choice of *τ*′ had a negligible effect (⩽2% change) on the estimated volume flow rates for *τ*′ ⩽ 10 ms. Thus, for the remainder of this paper we fixed *τ*′ at 1 ms.

### 4.2 Healthy volunteers

**Fig. 7:**
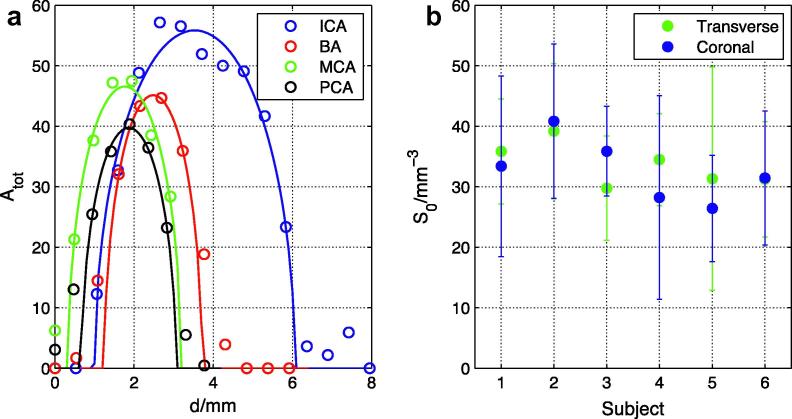
Signal calibration in healthy volunteers: (a) example *A*_tot_ values (circles) and fits to Eq. (4) (lines) in a number of arteries from one subject; and (b) mean and standard deviation of the calibration factor, *S*_0_, across all ten measurements for all subjects in both transverse and coronal views.

Calibration results in the healthy volunteers are summarized in Fig. 7. As for the flow phantom, the calibration equation (Eq. (4)) appears to describe the data well. There is considerable variation in the calculated calibration factor over the ten measurements made for each data set, but the mean values appear to be relatively consistent across subjects and between transverse and coronal views despite potential differences in coil loading and subject positioning. This suggests that the calibration procedure is reasonably robust and therefore is unlikely to lead to gross errors in the absolute volume flow rates which depend upon it.

**Fig. 8:**
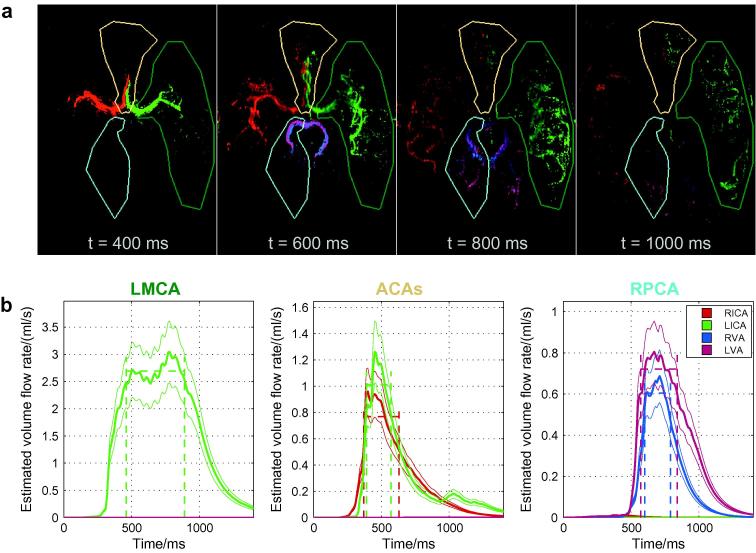
Examples of flow quantification in the transverse view of one subject: (a) selected frames of the simulated signal, *S*′, with overlaid masks of three example vessels (LMCA, ACAs and RPCA); and (b) the estimated blood volume flow rate over simulated time for each of the three masks. In the LMCA this estimate reaches a plateau as expected, giving confidence in the measurement, and similarly in the RPCA a small plateau region is present. However, in the ACAs the estimated flow rate never reaches a plateau and it is likely that the flow rate is underestimated in this case. The blood signal from each feeding artery is assigned a color in both the simulated signal images (a) and the flow rate plots (b) as shown in the legend. (For interpretation of the references to colour in this figure legend, the reader is referred to the web version of this article.)

Examples of flow rate quantification using transverse data from one healthy volunteer are given in Fig. 8. Note that by the time the simulated bolus reaches the circle of Willis there is already a considerable amount of dispersion which necessitates the use of large vessel masks to ensure the entire bolus is encompassed. In the LMCA the expected plateau behavior is observed, in a similar manner to that seen in the flow phantom, which suggests that the kinetic model is describing the data well, despite the gap between the imaging region and the labeling plane in the transverse view which leads to a more complex correction for RF attenuation effects. In the RPCA, which is fed approximately equally from both vertebral arteries in the subject shown, similar behavior is observed although the plateau region is quite small here. However, in the ACAs no plateau region is observed, meaning that it is likely that the simulated bolus starts to leave the imaging region, or the high signal thresholded mask, before the whole bolus has washed in. Thus, the simulated bolus never lies entirely within the vessel mask, implying that the flow rate is likely to be underestimated here.

**Fig. 9:**
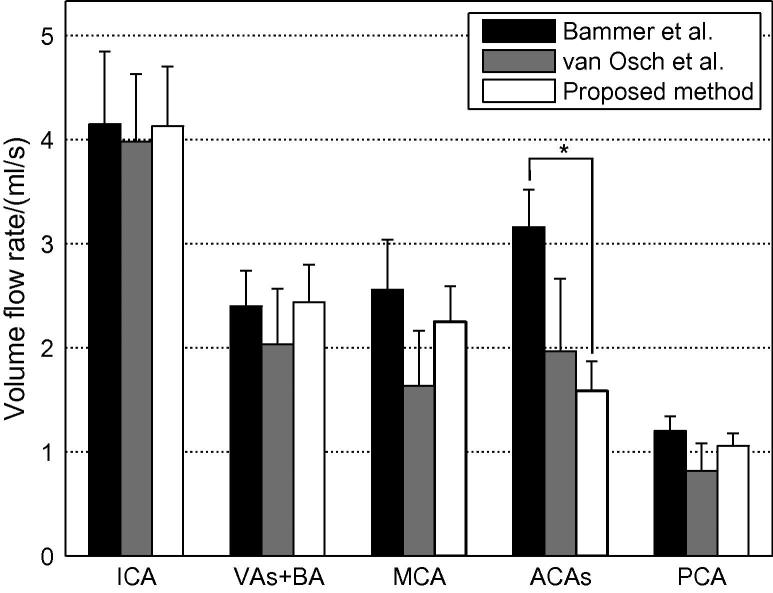
Mean estimated blood volume flow rates summed over feeding arteries and averaged across all subjects and views. Equivalent values in the same vessels as measured by Bammer et al. (2007) and van Osch et al. (2006) are also plotted for comparison. Error bars represent the standard error in the mean across subjects. Statistically significant differences (at the 0.05 level) are denoted with an asterisk (∗).

A summary of the estimated volume flow rates summed over all feeding arteries and averaged across all volunteers, using both coronal and transverse data sets, is given in Fig. 9. Values measured in healthy volunteers taken from studies by Bammer et al. (2007) (using phase-contrast MRA) and van Osch et al. (2006) (using pulsed ASL angiography) are plotted alongside for comparison. The values calculated using the proposed method generally agree well with those taken from these two papers. The only significant difference (as assessed using a two-tailed z-test with a significance level of 0.05) occurred in the ACAs (*p* = 0.0006) when compared to the measurements by van Osch et al. (2006).

Note that there are some differences in the measurement locations used in these studies. van Osch et al. (2006) quote ICA and BA flow rates based on the sum of flow rates in vessels distal to the circle of Willis (e.g. ICAs = MCAs + ACAs), assuming no collateral flow. In this study the flow rate in the VAs and BA was estimated together, whereas Bammer et al. (2007) performed the measurement in a cross-section of the BA. In addition, Bammer et al. (2007) estimated the flow rate in each ACA separately rather than together, so we have doubled the value quoted in their study to allow comparison here. However, these differences are expected to have only a small effect on the comparisons.

**Fig. 10:**
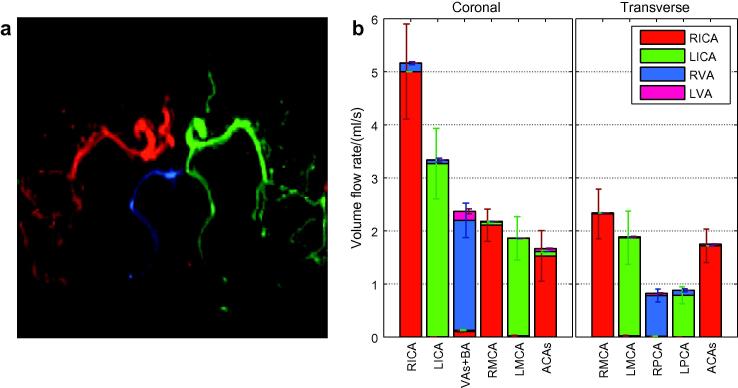
Example of the additional information gained by using a vessel-selective acquisition such as VEPCASL dynamic angiography in one healthy volunteer with non-standard flow patterns around the circle of Willis: (a) color-coded map of the relative blood volume parameter, *A*; and (b) the estimated volume flow rates in all vessel segments for this subject, in both coronal and transverse views, with the contribution from each feeding artery shown in a separate color (see legend). (For interpretation of the references to colour in this figure legend, the reader is referred to the web version of this article.)

The use of VEPCASL dynamic angiography means that not only can the total flow rate in a given vessel segment be quantified, but the contribution from each feeding artery can be assessed. An example of the potential utility of this additional information is given in Fig. 10. In this subject both ACAs are fed by the RICA, there is very little blood originating from the LVA and there is also considerable collateral flow from the LICA into the LPCA. The RICA flow rate is considerably higher than the LICA, perhaps since it is feeding both ACA territories, although the difference is not statistically significant. There is some minor contamination of signals due to background noise or motion artefacts (e.g. the presence of apparent RVA signal in the RICA).

In order to test consistency, the total blood volume flow rate entering the circle of Willis, as measured in the ICAs, VAs and BA, should be approximately equal to that flowing out, as measured in the MCAs, ACAs and PCAs, with the exception of the loss of some blood through small branching arteries such as those supplying the cerebellum, deep gray matter or the eyes. Across all subjects and feeding arteries (24 in total) there were only three cases in which there was a significant difference between the input and output flow rates (i.e. *p* < 0.05). One of these is the RVA of the subject shown in Fig. 10. On average output flow rates were lower than input flow rates by 0.6 ± 0.7 ml/s (mean ± standard deviation).

**Fig. 11:**
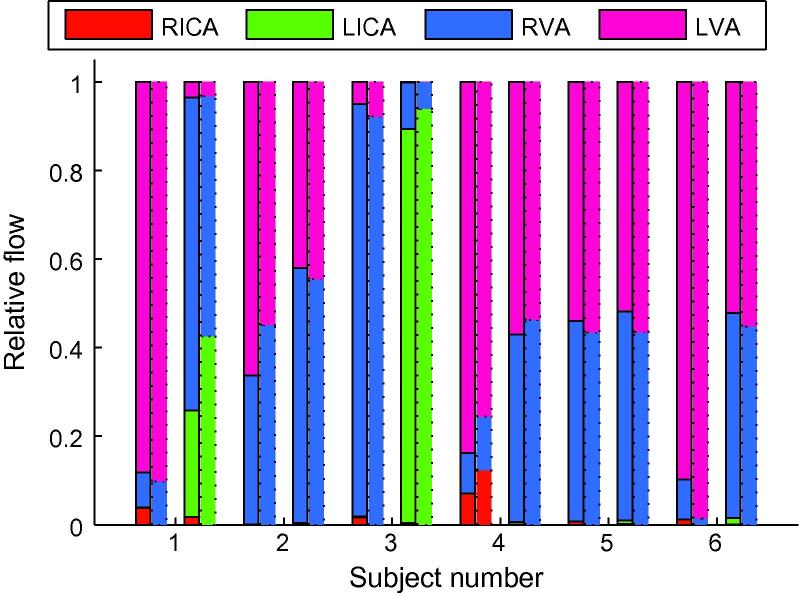
Comparison of relative blood volume flow rates from each feeding artery calculated using absolute flow rates (solid bars) or by summing *A* across a short vessel segment (dotted bars) in the PCAs of all subjects. For each subject the first pair of bars corresponds to the RPCA and the second pair to the LPCA. The relative contribution from each artery is color coded as before (see legend). (For interpretation of the references to colour in this figure legend, the reader is referred to the web version of this article.)

In a previous paper (Okell et al., 2012) we have shown that relative volume flow rates from each feeding artery can be calculated by summing the parameter *A* across small vessel masks in well-mixed downstream vessels. In the PCAs of the subjects used in this study, where considerable mixing of blood from feeding arteries occurs, there is good agreement between the relative flow rates calculated using this previous method, and the absolute flow rates measured for this study (see Fig. 11). The mean absolute difference between the two methods is 3 ± 4%.

## 5 Discussion

In this paper a novel method for blood volume flow rate quantification is described. This is likely to be of use in a number of situations, such as the identification of vascular territories that are at risk of ischemic damage in patients with steno-occlusive disease. This method is demonstrated using 2D vessel-encoded arterial spin labeling dynamic angiographic data, which provides vessel-specific information and requires no invasive procedure, ionizing radiation or contrast agent. However, this approach could also be used with other angiographic techniques, including those which are 3D.

The ability to calibrate the measured signal against blood volume using the same data set is a necessity for 2D projection methods such as X-ray DSA. It also removes the need for additional calibration scans, which is likely to be advantageous in a busy clinical protocol. For the 2D data sets considered here the calibration equation (Eq. (4)) appears to describe the data well in most cases (Figs. 6b and 7a), suggesting that the approximation of circular vessel cross-sections is reasonable, at least in the arteries of healthy volunteers considered here. However, a vessel with an elliptical cross-section would give a curve of the same shape but give an incorrect value for *S*_0_. This may explain some of the variability in the *S*_0_ estimates within each subject, but performing a sufficient number of calibration steps in different arteries should help to average out such effects. In addition, estimates of *S*_0_ could be skewed by incomplete filling of the arterial segment being used for calibration during the imaging time, leading to misestimation of the vessel diameter, although this should not be the case for the proximal vessel segments chosen in this study.

The fact that the calibration factor is fairly consistent across individuals and views (Fig. 7) suggests that the method is reasonably robust. This raises the possibility of using a single calibration factor for all subjects. However, variations in subject positioning within the coil, the coil loading and PCASL inversion efficiency could all affect the value of *S*_0_ appropriate for each subject, so a subject-specific calibration factor is likely to be more accurate.

In a flow phantom it was shown that the estimated volume flow rate evaluated over a range of simulated time points forms the expected plateau region (Fig. 6c). This indicates that the kinetic model describes well the signal attenuation due to *T*_1_ and RF effects since poor modeling of these factors would lead to an increasing or decreasing estimated flow rate over time. Averaging over this plateau region allows a more robust estimate of the flow rate to be obtained than taking a single time point alone. In addition, there is good agreement between the values estimated using this method and those measured directly using the flow phantom outflow pipe, particularly for flow rates below about 4.5 ml/s (Fig. 6d), which corresponds to those commonly found in the cerebral vasculature, with no significant systematic error (2 ± 3%).

The flow rate quantification appears to also work well in most of the cerebral vessels proximal and distal to the circle of Willis (see Fig. 8), yielding results that are generally consistent with the literature. Although results for only two other papers are plotted in Fig. 9, these are similar to those found in other studies (e.g. see Hendrikse et al., 2005 and Stock et al., 2000). However, the flow rate in the ACAs appears to be underestimated in our method compared to other studies. This is probably because branches of the ACAs were not captured within the imaging region and also because they are relatively small so might have been excluded from the high signal mask. Both of these factors mean the whole simulated bolus was not captured within the vessel mask so the flow rate was underestimated. In future work we plan to extend the imaging region used and improve the high signal masking to help overcome this problem.

The subject shown in Fig. 10 has a higher measured flow rate in the RICA than the LICA. This subject has a missing A1 segment in their left ACA, meaning that all the blood feeding both ACAs comes from the RICA. Subjects with this variant of the circle of Willis have been shown previously to have increased ICA flow rate on the contralateral side (Hendrikse et al., 2005). This perhaps contributed to the average RICA flow rate being higher than the LICA flow rate although the difference was not significant (*p* = 0.14).

The fact that the total blood flow rate distal to the circle of Willis was smaller than that measured in proximal vessels is not surprising, since some blood is diverted to the cerebellum, brainstem and deep gray matter before flowing into the main branches of the MCAs, ACAs and PCAs. The significant difference in a few cases, such as the RVA flow rate of the subject shown in Fig. 10, could also be due to not enough of the distal vessels being present in the imaging region for accurate quantification and/or exclusion of small vessel branches from the high signal mask.

Fig. 11 shows that our previous method (Okell et al., 2012) for calculating relative blood volume flow rates from each of the feeding arteries in well-mixed downstream vessels gives very similar results to the absolute quantification. This may be helpful in cases where this relative flow information is the main interest and a quick analysis is required or the vessel segment of interest is too short to encompass the dispersed simulated bolus.

To the best of our knowledge there is only one other study where quantitative blood volume flow rates have been derived from ASL-based angiographic data: van Osch et al. (2006) used a vessel-selective or non-selective pulsed ASL preparation followed by a segmented-EPI Look-Locker readout of a region encompassing the circle of Willis. Flow quantification was achieved using an arterial input function measured in a separate scan just proximal to the circle of Willis in combination with the summed signal across a vessel mask of interest distal to the circle of Willis. Accurate flow measurements were made with this method, despite imaging at only 1.5 T, and validated against phase-contrast angiographic data. However, that technique could not be applied directly to data acquired with continuous or pseudocontinuous labeling unless a short labeling duration was used and the labeling plane was positioned very proximally to ensure that the early part of the AIF could be measured. This would reduce the volume of labeled blood being produced and lead to greater *T*_1_ decay before the blood reaches the arteries of interest, leading to reduced SNR. In addition, this would restrict the optimal positioning of the labeling plane for vessel-encoding and potentially lead to problems with off-resonance effects further from the scanner isocentre.

Our proposed method has the advantages of being applicable to modalities in which such an absolute AIF cannot be measured and does not require the assumption of plug flow through the vessel mask of interest. However, the explicit modeling of the summed signal within the vessel mask enabled flow measurements to be made in smaller vessel segments than those used in our study. A similar extension to our method may be advantageous in the future.

The other commonly used method for flow rate quantification within arteries is phase-contrast MRA. Although 3D vector information can be obtained with this method and flow quantification performed across very short vessel segments, there is a lack of information regarding the arterial source of the flow. In addition, to achieve good coverage of the vessels scan times can be long: approximately 10 min to cover a 48 mm slab centered on the circle of Willis using a parallel imaging factor of three (Bammer et al., 2007). This would be considerably longer to extend coverage to allow assessment of the vertebral arteries also. Although the acquisition method presented in this paper is not very rapid (10 min per view), there is considerable scope for acceleration using parallel imaging (Pruessmann et al., 1999; Griswold et al., 2002), compressed sensing (Lustig et al., 2007) and the use of a balanced steady-state free precession readout (Okell et al., 2011). In addition, the analysis approach presented here is applicable to more rapid acquisition methods, such as X-ray DSA. Finally, partial volume effects can lead to the underestimation of flow rates in phase-contrast MRA (Bammer et al., 2007), particularly in small intra-cranial vessels. With ASL-based methods static tissue subtracts away, leaving a signal which only depends on the volume of blood present within the voxel, so partial volume effects do not affect the flow rate quantification.

It is worth noting that an alternative way to calculate the volume flow rate using our proposed method would be to simulate the signal using an infinite bolus duration. Once the simulated bolus enters the vessel mask of interest the slope of the summed signal across the mask could be used to calculate the volume flow rate. This is equivalent to the short simulated bolus method used in this study, although the identification of an appropriate region over which the flow rate can be measured is less obvious than identification of the plateau region used here.

Although only applied to VEPCASL dynamic angiography in this paper, the methodology described here could be used to quantify blood volume flow rates from a range of dynamic angiographic data sets, for example X-ray based data. The kinetic model used would need to be modified to account for the appropriate tracer input function, which could be predefined or measured, and signal attenuation over the course of the acquisition. However, the dispersion model, calibration method and use of a short simulated bolus should allow flow rate quantification irrespective of the underlying signal used, although further work is required to demonstrate this.

However, this study does have a number of limitations. The kinetic model used ignores the effect of flow pulsatility. This does not seem to adversely affect the measured flow rates in the cerebral vessels but would certainly be problematic in more pulsatile vessels. In addition, the choice of dispersion kernel becomes important when used to simulate a very short duration bolus. Although the gamma variate function used here appears to give a good fit to VEPCASL data (Okell et al., 2012), we have not investigated the use of alternative functions. However, in a recent study it has been shown that the choice of a gamma variate kernel gave the best fit to cerebral ASL angiographic data when compared to a range of other dispersion models (Chappell et al., 2013).

As discussed in the original paper (Okell et al., 2012), the CASL/PCASL kinetic model also makes the assumption that a single Δ*t*_min_ value is appropriate for the entire bolus. In reality there will be a range of arrival times due to laminar flow and other sources of bolus dispersion. This assumption only affects data acquired in the transverse view since the coronal data encompasses the labeling plane. The effect on the results presented here is likely to be minimal since the gap between the labeling plane and the imaging region is not very great so the majority of the bolus front should arrive in the imaging region at a similar time. Fits to the kinetic model did not appear to be adversely affected.

The calibration method for 2D data did not always provide a perfect fit to the data due to blurring of the vessel edges, perhaps due to the partial Fourier (asymmetric echo) readout method used or the presence of small perforating arteries branching off the main artery. Such effects are likely to be less significant when averaging over a number of vessel segments but could potentially bias the calibration factor estimate. It is expected that calibration using 3D data would be simpler and more robust.

In addition, this method requires the use of relatively large vessel masks to ensure that the entire dispersed simulated bolus is encompassed. This limits its utility in smaller arterial segments or where the arteries move outside the imaging region too quickly. One potential solution to this problem is to try to model the summed simulated signal within the vessel mask, in a manner similar to van Osch et al. (2006), to account for the whole bolus not being present within the mask at a given time. This would remove the need for the empirical method used to estimate the plateau region here, but would add an extra layer of complexity to the processing. The use of a high-signal mask may also exclude some smaller vessels, causing underestimation of flow rates, although it does speed up the processing time and removes some background noise.

Potentially, errors in fitting the kinetic model could affect the final calculated flow rate. However, uncertainties in the fitted parameter estimates are propagated through to the flow rate quantification so these should be accounted for. In addition, large numbers of voxels are used within each vessel mask so to some extent non-systematic errors will be averaged out.

Finally, the flow phantom measurements described here are somewhat idealized and use tubing with a fixed diameter. In vivo validation against a more established method, such as phase-contrast MRA, would be beneficial.

In future work we plan to extend this methodology by considering methods for improved identification of the plateau region, automated detection of whether the simulated bolus lies entirely within the mask and extension of the kinetic model to balanced steady-state free precession data (Okell et al., 2011). We hope to apply these methods to a range of data sets and in patients with vascular diseases.

## 6 Conclusions

The method described in this paper allows the quantification of blood volume flow rates using dynamic angiographic data. Its self-calibrating nature avoids the need for additional calibration scans. Results were presented from vessel-encoded arterial spin labeling data of the cerebral blood vessels, allowing the separate assessment of blood flow from each main brain-feeding artery. Validation was performed using a flow phantom, showing excellent agreement with the known flow rate, particularly below about 4.5 ml/s. Flow rate measurements in healthy volunteers agree well with the literature, except in the anterior cerebral arteries which were not contained sufficiently within the imaging region, although this could be remedied by extending the size of the imaging region in future experiments.

It is hoped that the quantitative information provided by this method could help with the assessment of collateral blood flow, treatment planning, and allow the more objective assessment of longitudinal changes to blood flow patterns over the course of a disease and the response to treatment.

## Figures and Tables

**Table 1:** A description of the mathematical symbols used in this paper. Where symbols differ from Okell et al. (2012) the equivalent is shown in square brackets. Symbols with a hat (ommitted here) represent estimated parameter values.

Symbol	Description
*Generic kinetic model parameters*	
*t*	Time
*S*(*t*)	Signal intensity in a given voxel
*c*_in_(*t*)	Relative tracer concentration in a proximal vessel
Δ*t*	Blood transit time from a proximal artery to the voxel [*δ*_*t*_]
*t*′	Time delay due to dispersion [*t*_*d*_]
*D*(*t*′)	Dispersion kernel
*T*(Δ*t*, *t*′,*t*)	Signal attenuation due to tracer decay
*Ψ*(Δ*t*, *t*′, *t*)	Signal attenuation due to the imaging method [*R*]
*v*	Volume of blood within vessels in the voxel
*S*_0_	Calibration factor: the signal intensity per unit blood volume
*A*	Scaling factor proportional to blood volume (*A* = *S*_0_*v*)

*Specific kinetic model parameters used in this paper*	
*τ*	VEPCASL labeling duration
*p*	Dispersion kernel time to peak
*s*	Dispersion kernel sharpness
Δ*t*_min_	Time at which labeled blood first arrives in the imaging region [*δ*_*t*,min_]

*Parameters used for calibration*	
*A*_tot_	Sum of *A* across all vascular components (if modeled separately)
Δ*x*, Δ*y*, Δ*z*	Voxel dimensions
$\overset{¯}{d}$	Vessel diameter
*d*	Distance perpendicular to the vessel direction
*d*_0_	Location of the center of the vessel

*Parameters used for flow rate quantification*	
*F*_*g*_	Volume flow rate of blood in vessel *g*
*V*_*g*_	Volume of labeled blood which flows into vessel *g*
*S*′	Signal simulated in the absence of attenuating effects
*τ*′	Simulated bolus duration
